# 3-Amino­benzoic acid–4,4′-bipyridine (2/3)

**DOI:** 10.1107/S1600536812033181

**Published:** 2012-07-28

**Authors:** Pornsuda Lhengwan, Supakit Achiwawanich, Tanwawan Duangthongyou

**Affiliations:** a Department of Chemistry, Faculty of Science, Kasetsart University, Bangkok 10903, Thailand

## Abstract

The asymmetric unit of the title compound, 3C_10_H_8_N_2_·2C_7_H_7_NO_2_, consists of three mol­ecules of 4,4′-bipyridine (bpy) and two mol­ecules of 3-amino­benzoic acid (bza). Two mol­ecules of bza and two mol­ecules of bpy are connected *via* O—H⋯N, N—H⋯N and N—H⋯O hydrogen bonds, forming forming infinite double-stranded zigzag chains along the *c* axis. The third mol­ecule of bpy is linked to the chain by weak C—H⋯O inter­actions. Adjacent chains are linked via π–π inter­actions [centroid–centroid distances = 3.759 (3)–3.928 (3) Å] involving the pyridine rings of bpy mol­ecules, resulting in a sheet-like structure parallel to (100). These sheets are stacked *via* C—H⋯π inter­actions, resulting finally in the formation of a three-dimensional supra­molecular structure.

## Related literature

For related structures, see: Karpova *et al.* (2004 ▶); Koteswara Rao *et al.* (2012 ▶); Yao *et al.* (2008 ▶); Zhao *et al.* (2007 ▶).
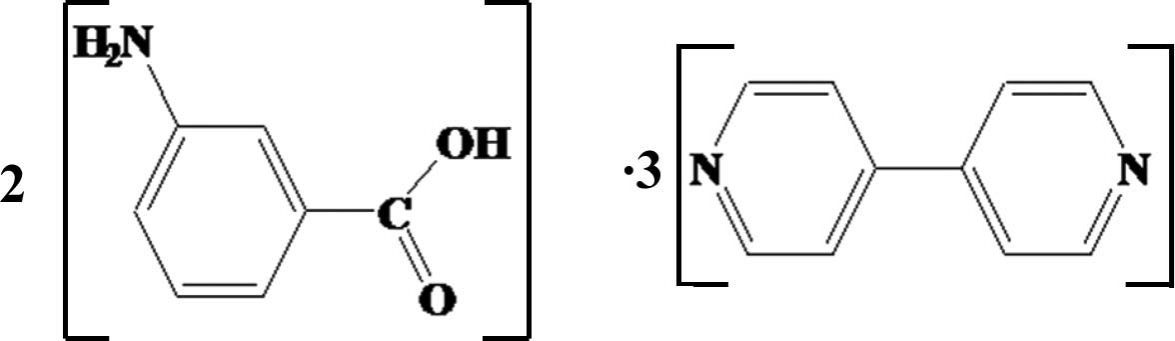


## Experimental

### 

#### Crystal data

3C_10_H_8_N_2_·2C_7_H_7_NO_2_
*M* *_r_* = 742.82
Triclinic, 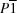

*a* = 9.371 (3) Å
*b* = 11.991 (4) Å
*c* = 17.653 (6) Å
α = 94.910 (11)°
β = 90.224 (10)°
γ = 102.128 (11)°
*V* = 1931.6 (11) Å^3^
*Z* = 2
Mo *K*α radiation
μ = 0.09 mm^−1^
*T* = 296 K
0.62 × 0.34 × 0.05 mm

#### Data collection

Bruker APEXII CCD diffractometer
14412 measured reflections
6518 independent reflections
3893 reflections with *I* > 2σ(*I*)
*R*_int_ = 0.025

#### Refinement

*R*[*F* ^2^ > 2σ(*F* ^2^)] = 0.071
*wR*(*F* ^2^) = 0.252
*S* = 1.07
6518 reflections
513 parameters
H atoms treated by a mixture of independent and constrained refinement
Δρ_max_ = 0.31 e Å^−3^
Δρ_min_ = −0.29 e Å^−3^

### 

Data collection: *APEX2* (Bruker, 2011 ▶); cell refinement: *SAINT* (Bruker, 2011 ▶); data reduction: *SAINT*; program(s) used to solve structure: *SHELXS97* (Sheldrick, 2008 ▶); program(s) used to refine structure: *SHELXL97* (Sheldrick, 2008 ▶); molecular graphics: *SHELXTL* (Sheldrick, 2008 ▶); software used to prepare material for publication: *SHELXTL*.

## Supplementary Material

Crystal structure: contains datablock(s) I, global. DOI: 10.1107/S1600536812033181/qm2077sup1.cif

Structure factors: contains datablock(s) I. DOI: 10.1107/S1600536812033181/qm2077Isup2.hkl

Supplementary material file. DOI: 10.1107/S1600536812033181/qm2077Isup3.cml

Additional supplementary materials:  crystallographic information; 3D view; checkCIF report

## Figures and Tables

**Table 1:** Hydrogen-bond geometry (Å, °) *Cg*7 and *Cg*8 are the centroids of the C31–C36 and C38–C43 rings, respectively.

*D*—H⋯*A*	*D*—H	H⋯*A*	*D*⋯*A*	*D*—H⋯*A*
O2—H′⋯N4	0.98 (4)	1.67 (4)	2.647 (4)	171 (3)
O4—H′′⋯N5	1.03 (5)	1.60 (5)	2.627 (4)	175 (4)
N7—H7*A*⋯N3^i^	0.86	2.16	3.007 (4)	169 (4)
N7—H7*B*⋯O3	0.86	2.23	3.038 (4)	156
N8—H8*A*⋯N6^ii^	0.86	2.16	3.006 (4)	169 (4)
N8—H8*B*⋯O1	0.86	2.21	3.019 (4)	158
C36—H36*A*⋯O3	0.93	2.52	3.300 (4)	141
C43—H43*A*⋯O1	0.93	2.53	3.304 (4)	141
C7—H7*C*⋯*Cg*7^iii^	0.93	2.87	3.751 (4)	157
C17—H17*A*⋯*Cg*8^iv^	0.93	2.72	3.562 (4)	151
C19—H19*A*⋯*Cg*8^v^	0.93	2.78	3.643 (4)	154
C22—H22*A*⋯*Cg*7^vi^	0.93	2.72	3.567 (4)	151

Symmetry codes: (i) 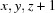; (ii) 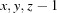; (iii) 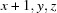; (iv) 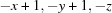; (v) 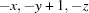; (vi) 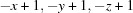.

**Fig. 1.**
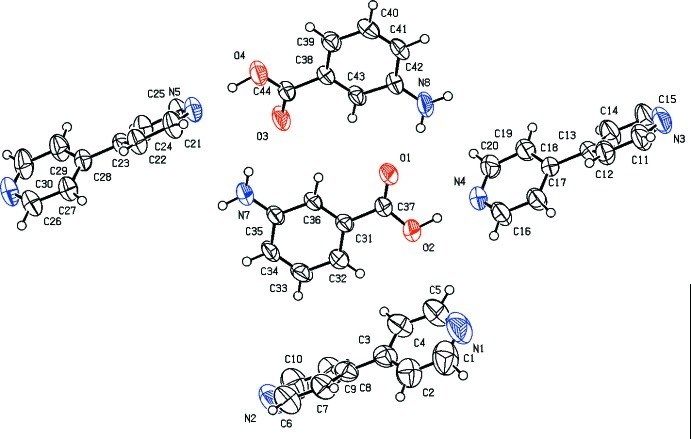
Molecular structure of the title compound (arbitrary spheres for the H atoms).

**Fig. 2.**
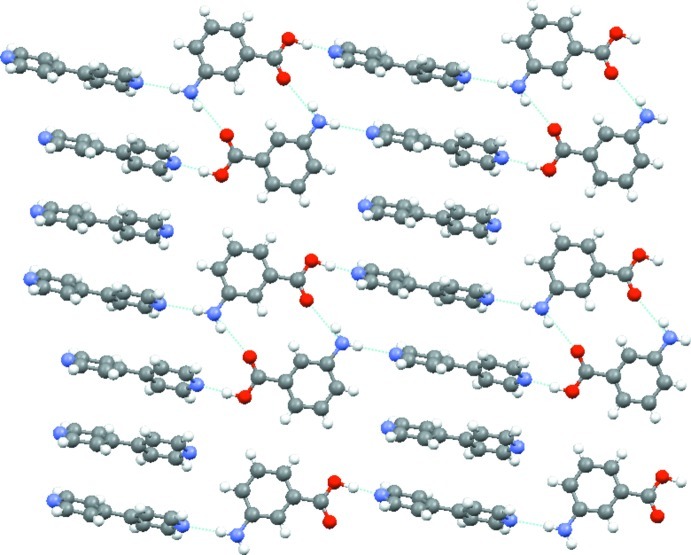
The two-dimensional-sheet structure showing π -π stacking (Hydrogen bonds indicated by dash lines).

**Fig. 3.**
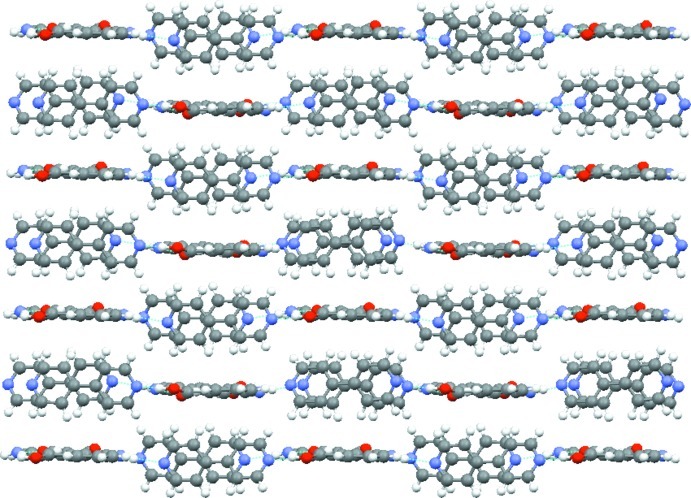
The three-dimensional-supramolecular structure showing the C-H to π interactions.

## supplementary crystallographic information

### Comment

The title compound was obtained as a side-product in the synthesis of metal-organic framework materials which similar to previeously observed organic compounds (Karpova *et al.* (2004); Koteswara Rao *et al.* (2012); Yao *et al.* (2008); Zhao *et al.* (2007). The asymmetric unit is composed of crystallographically independent of 4,4'-bipyridine (bpy) and 3-aminobenzoic acid (bza) in the stoichiometric ratio of 3:2 (Fig. 1). Three strong intermolecular hydrogen bonds are formed between bpy molecules and bza molecules *via* (1) amino and carboxylic acid groups of bza molecules (N—H···O), (2) amino groups of bza molecules and an N atom of bpy (N—H···N) and (3) a carboxylic group and an N atom of bpy (O—H···N), resulting in infinite double zigzag chain along *c*-axis (Table 1, Fig. 2) . The infinite chain is further stabilized by weak C—H···N interactions (distance 2.75 Å) with the third bpy molecule. In the chain the bpy and bza molecules are arranged in a nonplanar structure. Moreover the pyridine rings of bpy molecules exhibit a nonplanar configuration with dihedral angles in the range 28.9–32.66°. In addition, each of the infinite zigzag chains is linked to adjacent chains through π -π interactions between the pyridine rings of bpy molecules(distance 3.85 Å) forming a two-dimensional sheet sructure (Fig. 2). The two-dimensional– sheets are further connected through C—H···π interactions (distance 2.74 Å), resulting in a three-dimensional supramolecular structure (Fig. 3)

### Experimental

A solution of Mn(OAc)_2_.4H_2_O (1 mmol), 2-aminoterephthalic acid (1 mmol) and 4,4'-bipyridine(1 mmol) in 15 ml of H_2_O:DMF (1:1) was transferred into a Teflon lined aotoclave and heat at 170° for 15 h. Then, solution was slowly cooled to room temperature. Yellow crystral was obtained as a minor product from fragmentation of 2-aminoterephthalic acid to 3-aminobenzoic acid during reaction.

### Refinement

All H atoms of the compound were placed in the calculated positions with C—H =0.93 Å, N—H = 0.86 Å and included in the cycles of refinement in a rigid model, *U*_iso_(H) = 1.2 *U*_eq_(H). Except carboxylic acid H atom were located in different fourior map and restrained to their hosts.

### Crystal data

**Table:**

3C_10_H_8_N_2_·2C_7_H_7_NO_2_	*Z* = 2
*M**_r_* = 742.82	*F*(000) = 780
Triclinic, *P*1	*D*_x_ = 1.277 Mg m^−^^3^
*a* = 9.371 (3) Å	Mo *K*α radiation, λ = 0.71073 Å
*b* = 11.991 (4) Å	Cell parameters from 3914 reflections
*c* = 17.653 (6) Å	θ = 2.2–23.6°
α = 94.910 (11)°	µ = 0.09 mm^−^^1^
β = 90.224 (10)°	*T* = 296 K
γ = 102.128 (11)°	Plate, yellow
*V* = 1931.6 (11) Å^3^	0.62 × 0.34 × 0.05 mm

### Data collection

**Table:**

Bruker APEXII CCD diffractometer	3893 reflections with *I* > 2σ(*I*)
Radiation source: fine-focus sealed tube	*R*_int_ = 0.025
Graphite monochromator	θ_max_ = 25.0°, θ_min_ = 1.2°
φ and ω scans	*h* = −11→11
14412 measured reflections	*k* = −13→14
6518 independent reflections	*l* = −21→20

### Refinement

**Table:**

Refinement on *F*^2^	Primary atom site location: structure-invariant direct methods
Least-squares matrix: full	Secondary atom site location: difference Fourier map
*R*[*F*^2^ > 2σ(*F*^2^)] = 0.071	Hydrogen site location: inferred from neighbouring sites
*wR*(*F*^2^) = 0.252	H atoms treated by a mixture of independent and constrained refinement
*S* = 1.07	*w* = 1/[σ^2^(*F*_o_^2^) + (0.1384*P*)^2^ + 0.418*P*] where *P* = (*F*_o_^2^ + 2*F*_c_^2^)/3
6518 reflections	(Δ/σ)_max_ < 0.001
513 parameters	Δρ_max_ = 0.31 e Å^−^^3^
0 restraints	Δρ_min_ = −0.29 e Å^−^^3^

### Special details

**Table:**

Geometry. All e.s.d.'s (except the e.s.d. in the dihedral angle between two l.s. planes) are estimated using the full covariance matrix. The cell e.s.d.'s are taken into account individually in the estimation of e.s.d.'s in distances, angles and torsion angles; correlations between e.s.d.'s in cell parameters are only used when they are defined by crystal symmetry. An approximate (isotropic) treatment of cell e.s.d.'s is used for estimating e.s.d.'s involving l.s. planes.
Refinement. Refinement of *F*^2^ against ALL reflections. The weighted *R*-factor *wR* and goodness of fit *S* are based on *F*^2^, conventional *R*-factors *R* are based on *F*, with *F* set to zero for negative *F*^2^. The threshold expression of *F*^2^ > σ(*F*^2^) is used only for calculating *R*-factors(gt) *etc*. and is not relevant to the choice of reflections for refinement. *R*-factors based on *F*^2^ are statistically about twice as large as those based on *F*, and *R*- factors based on ALL data will be even larger.

### Fractional atomic coordinates and isotropic or equivalent isotropic displacement parameters (Å^2^)

**Table:**

	*x*	*y*	*z*	*U*_iso_*/*U*_eq_	
O4	0.2374 (3)	0.7665 (2)	0.37029 (13)	0.0653 (7)	
O2	0.2525 (3)	0.1595 (2)	0.12426 (13)	0.0686 (7)	
C38	0.2173 (3)	0.6986 (3)	0.24016 (15)	0.0419 (7)	
C31	0.2317 (3)	0.2219 (3)	0.25340 (16)	0.0453 (7)	
N7	0.2381 (3)	0.3911 (3)	0.43764 (14)	0.0716 (9)	
H7A	0.2429	0.3827	0.4854	0.086*	
H7B	0.2347	0.4570	0.4228	0.086*	
O3	0.1895 (3)	0.5792 (2)	0.34188 (12)	0.0728 (8)	
C35	0.2356 (3)	0.2994 (3)	0.38509 (16)	0.0480 (8)	
C41	0.2283 (3)	0.7337 (3)	0.08730 (17)	0.0527 (8)	
H41A	0.2319	0.7465	0.0361	0.063*	
C44	0.2138 (3)	0.6754 (3)	0.32162 (16)	0.0449 (7)	
O1	0.2077 (4)	0.3316 (2)	0.15097 (12)	0.0843 (9)	
C32	0.2392 (4)	0.1155 (3)	0.27634 (17)	0.0541 (8)	
H32A	0.2418	0.0545	0.2405	0.065*	
C18	0.2790 (3)	0.2763 (3)	−0.16668 (16)	0.0479 (8)	
C36	0.2291 (3)	0.3127 (3)	0.30716 (15)	0.0471 (8)	
H36A	0.2229	0.3835	0.2912	0.056*	
C43	0.2111 (3)	0.6072 (3)	0.18637 (15)	0.0461 (7)	
H43A	0.2019	0.5340	0.2021	0.055*	
C34	0.2409 (3)	0.1914 (3)	0.40706 (17)	0.0527 (8)	
H34A	0.2433	0.1802	0.4585	0.063*	
N8	0.2155 (4)	0.5309 (3)	0.05628 (14)	0.0773 (10)	
H8A	0.2199	0.5406	0.0086	0.093*	
H8B	0.2094	0.4636	0.0711	0.093*	
C23	0.2592 (3)	0.6558 (3)	0.66064 (16)	0.0473 (7)	
C42	0.2184 (3)	0.6223 (3)	0.10845 (16)	0.0491 (8)	
C37	0.2289 (3)	0.2435 (3)	0.17188 (16)	0.0500 (8)	
C13	0.2825 (3)	0.3010 (3)	−0.24750 (16)	0.0497 (8)	
C39	0.2286 (3)	0.8082 (3)	0.21794 (17)	0.0514 (8)	
H39A	0.2332	0.8702	0.2541	0.062*	
N6	0.2662 (4)	0.5891 (3)	0.89493 (15)	0.0756 (9)	
C28	0.2610 (3)	0.6319 (3)	0.74141 (16)	0.0479 (8)	
N4	0.2684 (3)	0.2220 (3)	−0.01622 (15)	0.0640 (8)	
N3	0.2904 (4)	0.3476 (3)	−0.40037 (16)	0.0818 (10)	
N5	0.2546 (3)	0.7082 (3)	0.50962 (14)	0.0612 (8)	
C33	0.2426 (4)	0.1014 (3)	0.35363 (18)	0.0579 (9)	
H33A	0.2461	0.0300	0.3694	0.070*	
C40	0.2329 (3)	0.8241 (3)	0.14098 (18)	0.0554 (8)	
H40A	0.2391	0.8972	0.1256	0.067*	
C27	0.3880 (4)	0.6226 (3)	0.77845 (17)	0.0606 (9)	
H27A	0.4750	0.6300	0.7523	0.073*	
C26	0.3852 (4)	0.6022 (4)	0.85425 (19)	0.0717 (11)	
H26A	0.4722	0.5975	0.8780	0.086*	
C17	0.3960 (4)	0.2479 (3)	−0.13103 (18)	0.0626 (10)	
H17A	0.4814	0.2462	−0.1574	0.075*	
C19	0.1573 (4)	0.2791 (3)	−0.12358 (18)	0.0623 (9)	
H19A	0.0758	0.2989	−0.1445	0.075*	
C24	0.1354 (4)	0.6189 (3)	0.61445 (18)	0.0615 (9)	
H24A	0.0510	0.5757	0.6334	0.074*	
C12	0.4107 (4)	0.3535 (3)	−0.28055 (18)	0.0643 (10)	
H12A	0.4973	0.3738	−0.2520	0.077*	
C22	0.3807 (4)	0.7180 (3)	0.62811 (18)	0.0619 (9)	
H22A	0.4668	0.7438	0.6566	0.074*	
C16	0.3865 (4)	0.2222 (4)	−0.05703 (18)	0.0704 (11)	
H16A	0.4673	0.2039	−0.0342	0.084*	
C25	0.1382 (4)	0.6465 (3)	0.54070 (18)	0.0665 (10)	
H25A	0.0541	0.6206	0.5106	0.080*	
C11	0.4087 (5)	0.3751 (4)	−0.35570 (19)	0.0762 (11)	
H11A	0.4955	0.4112	−0.3764	0.091*	
C21	0.3740 (4)	0.7417 (3)	0.55334 (18)	0.0675 (10)	
H21A	0.4574	0.7832	0.5325	0.081*	
C20	0.1572 (4)	0.2523 (3)	−0.04944 (19)	0.0685 (10)	
H20A	0.0746	0.2558	−0.0211	0.082*	
C14	0.1599 (4)	0.2723 (4)	−0.29407 (19)	0.0750 (11)	
H14A	0.0711	0.2366	−0.2751	0.090*	
C29	0.1364 (4)	0.6188 (4)	0.78445 (18)	0.0706 (11)	
H29A	0.0476	0.6237	0.7628	0.085*	
C30	0.1453 (5)	0.5984 (4)	0.8596 (2)	0.0818 (12)	
H30A	0.0603	0.5907	0.8875	0.098*	
C15	0.1693 (5)	0.2968 (5)	−0.3688 (2)	0.0928 (15)	
H15A	0.0849	0.2761	−0.3991	0.111*	
C8	0.7232 (4)	0.0224 (3)	0.2943 (2)	0.0598 (9)	
C3	0.7286 (4)	0.0451 (3)	0.2136 (2)	0.0647 (9)	
C9	0.5967 (4)	−0.0339 (3)	0.3258 (2)	0.0760 (11)	
H9A	0.5130	−0.0604	0.2955	0.091*	
N2	0.7093 (6)	−0.0184 (4)	0.4481 (2)	0.1066 (13)	
C4	0.6089 (5)	0.0650 (4)	0.1757 (2)	0.0874 (13)	
H4B	0.5225	0.0660	0.2012	0.105*	
C7	0.8441 (4)	0.0586 (4)	0.3433 (2)	0.0790 (11)	
H7C	0.9323	0.0976	0.3255	0.095*	
N1	0.7348 (8)	0.0833 (4)	0.0594 (2)	0.1276 (18)	
C10	0.5937 (6)	−0.0509 (4)	0.4012 (3)	0.0995 (15)	
H10A	0.5060	−0.0874	0.4210	0.119*	
C2	0.8530 (5)	0.0441 (4)	0.1716 (2)	0.0866 (12)	
H2B	0.9366	0.0303	0.1943	0.104*	
C6	0.8314 (6)	0.0361 (5)	0.4181 (3)	0.0984 (15)	
H6B	0.9135	0.0604	0.4498	0.118*	
C5	0.6177 (8)	0.0833 (5)	0.1007 (3)	0.122 (2)	
H5B	0.5351	0.0968	0.0768	0.146*	
C1	0.8513 (7)	0.0639 (5)	0.0957 (3)	0.1154 (18)	
H1B	0.9359	0.0637	0.0684	0.138*	
H''	0.239 (4)	0.744 (4)	0.425 (2)	0.101 (13)*	
H'	0.248 (4)	0.178 (4)	0.071 (2)	0.099 (13)*	

### Atomic displacement parameters (Å^2^)

**Table:**

	*U*^11^	*U*^22^	*U*^33^	*U*^12^	*U*^13^	*U*^23^
O4	0.1013 (18)	0.0551 (15)	0.0357 (12)	0.0066 (13)	−0.0032 (12)	0.0073 (11)
O2	0.1079 (19)	0.0677 (17)	0.0377 (13)	0.0335 (14)	0.0087 (12)	0.0097 (12)
C38	0.0456 (16)	0.0447 (18)	0.0357 (15)	0.0066 (13)	−0.0013 (12)	0.0125 (14)
C31	0.0505 (17)	0.0510 (19)	0.0385 (16)	0.0160 (14)	0.0045 (13)	0.0141 (14)
N7	0.114 (2)	0.076 (2)	0.0328 (14)	0.0344 (19)	−0.0027 (15)	0.0096 (15)
O3	0.130 (2)	0.0503 (15)	0.0366 (12)	0.0117 (14)	0.0021 (12)	0.0158 (11)
C35	0.0530 (18)	0.061 (2)	0.0345 (15)	0.0193 (15)	0.0021 (13)	0.0147 (15)
C41	0.0566 (19)	0.066 (2)	0.0381 (16)	0.0107 (16)	−0.0010 (13)	0.0227 (16)
C44	0.0517 (17)	0.0478 (19)	0.0350 (15)	0.0080 (14)	0.0001 (12)	0.0087 (15)
O1	0.161 (3)	0.0675 (17)	0.0400 (13)	0.0535 (18)	0.0096 (14)	0.0191 (13)
C32	0.069 (2)	0.051 (2)	0.0467 (18)	0.0176 (16)	0.0069 (15)	0.0143 (16)
C18	0.0554 (18)	0.054 (2)	0.0376 (16)	0.0186 (15)	0.0016 (14)	0.0050 (14)
C36	0.0579 (18)	0.0513 (19)	0.0369 (16)	0.0178 (15)	0.0026 (13)	0.0154 (14)
C43	0.0595 (18)	0.0426 (18)	0.0358 (15)	0.0053 (14)	0.0009 (13)	0.0152 (14)
C34	0.0572 (19)	0.072 (2)	0.0343 (15)	0.0181 (16)	0.0030 (13)	0.0229 (16)
N8	0.139 (3)	0.064 (2)	0.0304 (14)	0.0217 (19)	0.0057 (16)	0.0099 (14)
C23	0.0546 (18)	0.0477 (19)	0.0389 (16)	0.0070 (15)	0.0030 (14)	0.0093 (14)
C42	0.0566 (18)	0.057 (2)	0.0332 (15)	0.0070 (15)	0.0004 (13)	0.0123 (15)
C37	0.066 (2)	0.051 (2)	0.0363 (16)	0.0167 (16)	0.0026 (14)	0.0084 (15)
C13	0.0586 (19)	0.060 (2)	0.0368 (16)	0.0263 (16)	0.0008 (14)	0.0084 (15)
C39	0.063 (2)	0.0462 (19)	0.0448 (17)	0.0091 (15)	−0.0030 (14)	0.0090 (15)
N6	0.091 (2)	0.094 (3)	0.0376 (15)	0.0057 (19)	0.0053 (16)	0.0166 (16)
C28	0.0571 (19)	0.0473 (19)	0.0356 (16)	0.0021 (14)	0.0008 (14)	0.0054 (14)
N4	0.081 (2)	0.078 (2)	0.0371 (14)	0.0252 (16)	0.0047 (14)	0.0070 (14)
N3	0.097 (3)	0.115 (3)	0.0423 (16)	0.036 (2)	0.0021 (17)	0.0206 (18)
N5	0.078 (2)	0.0669 (19)	0.0357 (14)	0.0062 (15)	−0.0010 (14)	0.0074 (14)
C33	0.068 (2)	0.060 (2)	0.054 (2)	0.0214 (17)	0.0041 (16)	0.0254 (18)
C40	0.066 (2)	0.049 (2)	0.054 (2)	0.0092 (16)	−0.0025 (15)	0.0241 (17)
C27	0.066 (2)	0.072 (2)	0.0423 (18)	0.0084 (18)	0.0042 (15)	0.0138 (17)
C26	0.083 (3)	0.083 (3)	0.047 (2)	0.010 (2)	−0.0066 (19)	0.0183 (19)
C17	0.059 (2)	0.092 (3)	0.0444 (18)	0.0281 (19)	0.0010 (15)	0.0155 (18)
C19	0.064 (2)	0.084 (3)	0.0465 (18)	0.0295 (19)	0.0041 (16)	0.0138 (18)
C24	0.061 (2)	0.073 (2)	0.0446 (18)	−0.0025 (17)	0.0021 (15)	0.0102 (17)
C12	0.064 (2)	0.088 (3)	0.0443 (18)	0.0205 (19)	0.0021 (16)	0.0125 (18)
C22	0.058 (2)	0.082 (3)	0.0429 (18)	0.0045 (18)	−0.0003 (15)	0.0156 (18)
C16	0.069 (2)	0.105 (3)	0.0449 (19)	0.030 (2)	−0.0025 (17)	0.023 (2)
C25	0.071 (2)	0.079 (3)	0.0443 (19)	0.003 (2)	−0.0107 (16)	0.0053 (18)
C11	0.086 (3)	0.101 (3)	0.047 (2)	0.025 (2)	0.0108 (19)	0.024 (2)
C21	0.068 (2)	0.086 (3)	0.0453 (19)	0.0020 (19)	0.0060 (17)	0.0181 (19)
C20	0.075 (2)	0.092 (3)	0.0468 (19)	0.033 (2)	0.0143 (17)	0.0145 (19)
C14	0.061 (2)	0.119 (4)	0.050 (2)	0.024 (2)	−0.0042 (17)	0.020 (2)
C29	0.063 (2)	0.104 (3)	0.0454 (19)	0.015 (2)	0.0073 (16)	0.019 (2)
C30	0.081 (3)	0.117 (4)	0.045 (2)	0.011 (2)	0.0157 (19)	0.014 (2)
C15	0.081 (3)	0.150 (5)	0.051 (2)	0.030 (3)	−0.015 (2)	0.021 (3)
C8	0.066 (2)	0.048 (2)	0.067 (2)	0.0104 (17)	−0.0014 (17)	0.0148 (17)
C3	0.077 (2)	0.046 (2)	0.070 (2)	0.0068 (17)	0.000 (2)	0.0151 (18)
C9	0.075 (3)	0.067 (3)	0.079 (3)	−0.006 (2)	−0.006 (2)	0.020 (2)
N2	0.140 (4)	0.104 (3)	0.079 (3)	0.019 (3)	−0.002 (3)	0.041 (2)
C4	0.111 (3)	0.086 (3)	0.074 (3)	0.036 (3)	−0.006 (2)	0.022 (2)
C7	0.069 (2)	0.090 (3)	0.078 (3)	0.012 (2)	−0.005 (2)	0.019 (2)
N1	0.213 (6)	0.098 (3)	0.074 (3)	0.027 (4)	0.007 (3)	0.035 (3)
C10	0.110 (4)	0.095 (4)	0.087 (3)	−0.004 (3)	0.008 (3)	0.036 (3)
C2	0.089 (3)	0.083 (3)	0.080 (3)	−0.003 (2)	0.009 (2)	0.015 (2)
C6	0.100 (4)	0.117 (4)	0.080 (3)	0.023 (3)	−0.020 (3)	0.019 (3)
C5	0.179 (6)	0.121 (5)	0.081 (4)	0.062 (4)	−0.014 (4)	0.029 (3)
C1	0.150 (5)	0.103 (4)	0.082 (3)	−0.003 (4)	0.030 (3)	0.022 (3)

### Geometric parameters (Å, º)

**Table:**

O4—C44	1.310 (4)	N5—C25	1.332 (5)
O4—H''	1.03 (4)	C33—H33A	0.9300
O2—C37	1.312 (4)	C40—H40A	0.9300
O2—H'	0.98 (4)	C27—C26	1.380 (4)
C38—C43	1.378 (4)	C27—H27A	0.9300
C38—C39	1.387 (4)	C26—H26A	0.9300
C38—C44	1.487 (4)	C17—C16	1.367 (4)
C31—C32	1.386 (4)	C17—H17A	0.9300
C31—C36	1.386 (4)	C19—C20	1.374 (4)
C31—C37	1.485 (4)	C19—H19A	0.9300
N7—C35	1.373 (4)	C24—C25	1.369 (5)
N7—H7A	0.8600	C24—H24A	0.9300
N7—H7B	0.8600	C12—C11	1.374 (5)
O3—C44	1.213 (4)	C12—H12A	0.9300
C35—C34	1.394 (4)	C22—C21	1.378 (4)
C35—C36	1.401 (4)	C22—H22A	0.9300
C41—C40	1.370 (4)	C16—H16A	0.9300
C41—C42	1.402 (5)	C25—H25A	0.9300
C41—H41A	0.9300	C11—H11A	0.9300
O1—C37	1.203 (4)	C21—H21A	0.9300
C32—C33	1.390 (4)	C20—H20A	0.9300
C32—H32A	0.9300	C14—C15	1.375 (5)
C18—C19	1.378 (4)	C14—H14A	0.9300
C18—C17	1.379 (4)	C29—C30	1.374 (5)
C18—C13	1.481 (4)	C29—H29A	0.9300
C36—H36A	0.9300	C30—H30A	0.9300
C43—C42	1.403 (4)	C15—H15A	0.9300
C43—H43A	0.9300	C8—C9	1.380 (5)
C34—C33	1.374 (4)	C8—C7	1.393 (5)
C34—H34A	0.9300	C8—C3	1.472 (5)
N8—C42	1.365 (4)	C3—C4	1.378 (5)
N8—H8A	0.8600	C3—C2	1.385 (6)
N8—H8B	0.8600	C9—C10	1.363 (6)
C23—C22	1.380 (4)	C9—H9A	0.9300
C23—C24	1.386 (4)	N2—C6	1.329 (6)
C23—C28	1.479 (4)	N2—C10	1.331 (6)
C13—C14	1.376 (4)	C4—C5	1.359 (6)
C13—C12	1.389 (5)	C4—H4B	0.9300
C39—C40	1.387 (4)	C7—C6	1.370 (6)
C39—H39A	0.9300	C7—H7C	0.9300
N6—C26	1.317 (5)	N1—C5	1.320 (7)
N6—C30	1.322 (5)	N1—C1	1.336 (7)
C28—C29	1.385 (4)	C10—H10A	0.9300
C28—C27	1.385 (4)	C2—C1	1.381 (6)
N4—C16	1.323 (4)	C2—H2B	0.9300
N4—C20	1.324 (4)	C6—H6B	0.9300
N3—C15	1.320 (5)	C5—H5B	0.9300
N3—C11	1.324 (5)	C1—H1B	0.9300
N5—C21	1.327 (4)		
			
C44—O4—H''	111 (2)	C16—C17—H17A	120.0
C37—O2—H'	111 (2)	C18—C17—H17A	120.0
C43—C38—C39	120.3 (3)	C20—C19—C18	119.5 (3)
C43—C38—C44	117.9 (3)	C20—C19—H19A	120.2
C39—C38—C44	121.9 (3)	C18—C19—H19A	120.2
C32—C31—C36	120.1 (3)	C25—C24—C23	119.5 (3)
C32—C31—C37	122.1 (3)	C25—C24—H24A	120.2
C36—C31—C37	117.8 (3)	C23—C24—H24A	120.2
C35—N7—H7A	120.0	C11—C12—C13	119.5 (3)
C35—N7—H7B	120.0	C11—C12—H12A	120.2
H7A—N7—H7B	120.0	C13—C12—H12A	120.2
N7—C35—C34	121.6 (3)	C21—C22—C23	119.8 (3)
N7—C35—C36	120.4 (3)	C21—C22—H22A	120.1
C34—C35—C36	118.0 (3)	C23—C22—H22A	120.1
C40—C41—C42	121.0 (3)	N4—C16—C17	123.3 (3)
C40—C41—H41A	119.5	N4—C16—H16A	118.3
C42—C41—H41A	119.5	C17—C16—H16A	118.3
O3—C44—O4	122.1 (3)	N5—C25—C24	123.7 (3)
O3—C44—C38	122.7 (3)	N5—C25—H25A	118.2
O4—C44—C38	115.2 (3)	C24—C25—H25A	118.2
C31—C32—C33	119.1 (3)	N3—C11—C12	123.9 (4)
C31—C32—H32A	120.5	N3—C11—H11A	118.0
C33—C32—H32A	120.5	C12—C11—H11A	118.0
C19—C18—C17	116.6 (3)	N5—C21—C22	123.3 (3)
C19—C18—C13	121.4 (3)	N5—C21—H21A	118.4
C17—C18—C13	122.0 (3)	C22—C21—H21A	118.4
C31—C36—C35	121.0 (3)	N4—C20—C19	123.5 (3)
C31—C36—H36A	119.5	N4—C20—H20A	118.2
C35—C36—H36A	119.5	C19—C20—H20A	118.2
C38—C43—C42	121.4 (3)	C15—C14—C13	119.6 (4)
C38—C43—H43A	119.3	C15—C14—H14A	120.2
C42—C43—H43A	119.3	C13—C14—H14A	120.2
C33—C34—C35	120.8 (3)	C30—C29—C28	119.3 (3)
C33—C34—H34A	119.6	C30—C29—H29A	120.3
C35—C34—H34A	119.6	C28—C29—H29A	120.3
C42—N8—H8A	120.0	N6—C30—C29	124.5 (4)
C42—N8—H8B	120.0	N6—C30—H30A	117.7
H8A—N8—H8B	120.0	C29—C30—H30A	117.7
C22—C23—C24	116.8 (3)	N3—C15—C14	124.3 (4)
C22—C23—C28	121.2 (3)	N3—C15—H15A	117.9
C24—C23—C28	121.9 (3)	C14—C15—H15A	117.9
N8—C42—C41	122.3 (3)	C9—C8—C7	116.4 (4)
N8—C42—C43	120.4 (3)	C9—C8—C3	121.6 (3)
C41—C42—C43	117.3 (3)	C7—C8—C3	122.0 (3)
O1—C37—O2	122.4 (3)	C4—C3—C2	116.8 (4)
O1—C37—C31	122.7 (3)	C4—C3—C8	121.6 (4)
O2—C37—C31	114.9 (3)	C2—C3—C8	121.5 (4)
C14—C13—C12	116.4 (3)	C10—C9—C8	120.3 (4)
C14—C13—C18	122.0 (3)	C10—C9—H9A	119.8
C12—C13—C18	121.6 (3)	C8—C9—H9A	119.8
C38—C39—C40	118.9 (3)	C6—N2—C10	116.2 (4)
C38—C39—H39A	120.5	C5—C4—C3	119.7 (5)
C40—C39—H39A	120.5	C5—C4—H4B	120.2
C26—N6—C30	116.3 (3)	C3—C4—H4B	120.2
C29—C28—C27	116.3 (3)	C6—C7—C8	119.2 (4)
C29—C28—C23	121.9 (3)	C6—C7—H7C	120.4
C27—C28—C23	121.8 (3)	C8—C7—H7C	120.4
C16—N4—C20	116.9 (3)	C5—N1—C1	115.9 (5)
C15—N3—C11	116.2 (3)	N2—C10—C9	123.7 (5)
C21—N5—C25	116.8 (3)	N2—C10—H10A	118.2
C34—C33—C32	121.0 (3)	C9—C10—H10A	118.2
C34—C33—H33A	119.5	C1—C2—C3	119.2 (5)
C32—C33—H33A	119.5	C1—C2—H2B	120.4
C41—C40—C39	121.0 (3)	C3—C2—H2B	120.4
C41—C40—H40A	119.5	N2—C6—C7	124.2 (4)
C39—C40—H40A	119.5	N2—C6—H6B	117.9
C26—C27—C28	119.8 (3)	C7—C6—H6B	117.9
C26—C27—H27A	120.1	N1—C5—C4	124.8 (5)
C28—C27—H27A	120.1	N1—C5—H5B	117.6
N6—C26—C27	123.8 (3)	C4—C5—H5B	117.6
N6—C26—H26A	118.1	N1—C1—C2	123.6 (6)
C27—C26—H26A	118.1	N1—C1—H1B	118.2
C16—C17—C18	120.0 (3)	C2—C1—H1B	118.2
			
C43—C38—C44—O3	−8.5 (4)	C22—C23—C24—C25	−1.0 (5)
C39—C38—C44—O3	172.5 (3)	C28—C23—C24—C25	178.0 (3)
C43—C38—C44—O4	172.2 (3)	C14—C13—C12—C11	0.9 (5)
C39—C38—C44—O4	−6.8 (4)	C18—C13—C12—C11	−179.8 (3)
C36—C31—C32—C33	0.5 (5)	C24—C23—C22—C21	0.9 (5)
C37—C31—C32—C33	179.5 (3)	C28—C23—C22—C21	−178.1 (3)
C32—C31—C36—C35	0.8 (4)	C20—N4—C16—C17	−2.4 (6)
C37—C31—C36—C35	−178.2 (3)	C18—C17—C16—N4	0.5 (6)
N7—C35—C36—C31	177.7 (3)	C21—N5—C25—C24	1.5 (6)
C34—C35—C36—C31	−1.7 (4)	C23—C24—C25—N5	−0.2 (6)
C39—C38—C43—C42	1.1 (4)	C15—N3—C11—C12	0.1 (6)
C44—C38—C43—C42	−178.0 (3)	C13—C12—C11—N3	−0.8 (6)
N7—C35—C34—C33	−178.1 (3)	C25—N5—C21—C22	−1.6 (6)
C36—C35—C34—C33	1.3 (4)	C23—C22—C21—N5	0.4 (6)
C40—C41—C42—N8	−179.1 (3)	C16—N4—C20—C19	2.6 (6)
C40—C41—C42—C43	1.0 (4)	C18—C19—C20—N4	−0.9 (6)
C38—C43—C42—N8	178.5 (3)	C12—C13—C14—C15	−0.4 (6)
C38—C43—C42—C41	−1.7 (4)	C18—C13—C14—C15	−179.7 (4)
C32—C31—C37—O1	172.3 (3)	C27—C28—C29—C30	0.3 (6)
C36—C31—C37—O1	−8.8 (5)	C23—C28—C29—C30	−179.0 (4)
C32—C31—C37—O2	−8.2 (4)	C26—N6—C30—C29	1.0 (7)
C36—C31—C37—O2	170.7 (3)	C28—C29—C30—N6	−0.6 (7)
C19—C18—C13—C14	−33.3 (5)	C11—N3—C15—C14	0.5 (7)
C17—C18—C13—C14	145.8 (4)	C13—C14—C15—N3	−0.3 (7)
C19—C18—C13—C12	147.5 (4)	C9—C8—C3—C4	32.3 (6)
C17—C18—C13—C12	−33.4 (5)	C7—C8—C3—C4	−146.3 (4)
C43—C38—C39—C40	0.3 (4)	C9—C8—C3—C2	−145.9 (4)
C44—C38—C39—C40	179.3 (3)	C7—C8—C3—C2	35.6 (6)
C22—C23—C28—C29	150.1 (4)	C7—C8—C9—C10	0.5 (6)
C24—C23—C28—C29	−28.8 (5)	C3—C8—C9—C10	−178.2 (4)
C22—C23—C28—C27	−29.2 (5)	C2—C3—C4—C5	−0.5 (7)
C24—C23—C28—C27	151.9 (3)	C8—C3—C4—C5	−178.7 (4)
C35—C34—C33—C32	0.0 (5)	C9—C8—C7—C6	0.5 (6)
C31—C32—C33—C34	−0.9 (5)	C3—C8—C7—C6	179.2 (4)
C42—C41—C40—C39	0.3 (5)	C6—N2—C10—C9	1.7 (8)
C38—C39—C40—C41	−0.9 (5)	C8—C9—C10—N2	−1.7 (8)
C29—C28—C27—C26	−0.5 (5)	C4—C3—C2—C1	0.6 (6)
C23—C28—C27—C26	178.8 (3)	C8—C3—C2—C1	178.9 (4)
C30—N6—C26—C27	−1.2 (6)	C10—N2—C6—C7	−0.6 (8)
C28—C27—C26—N6	1.0 (6)	C8—C7—C6—N2	−0.5 (8)
C19—C18—C17—C16	1.2 (5)	C1—N1—C5—C4	−0.2 (9)
C13—C18—C17—C16	−178.0 (3)	C3—C4—C5—N1	0.3 (9)
C17—C18—C19—C20	−1.0 (5)	C5—N1—C1—C2	0.4 (9)
C13—C18—C19—C20	178.1 (3)	C3—C2—C1—N1	−0.6 (8)

### Hydrogen-bond geometry (Å, º)

Cg7 and Cg8 are the centroids of the C31–C36 and C38–C43 rings, respectively.

**Table:**

*D*—H···*A*	*D*—H	H···*A*	*D*···*A*	*D*—H···*A*
O2—H′···N4	0.98 (4)	1.67 (4)	2.647 (4)	171 (3)
O4—H′′···N5	1.03 (5)	1.60 (5)	2.627 (4)	175 (4)
N7—H7*A*···N3^i^	0.86	2.16	3.007 (4)	169 (4)
N7—H7*B*···O3	0.86	2.23	3.038 (4)	156
N8—H8*A*···N6^ii^	0.86	2.16	3.006 (4)	169 (4)
N8—H8*B*···O1	0.86	2.21	3.019 (4)	158
C36—H36*A*···O3	0.93	2.52	3.300 (4)	141
C43—H43*A*···O1	0.93	2.53	3.304 (4)	141
C7—H7*C*···*Cg*7^iii^	0.93	2.87	3.751 (4)	157
C17—H17*A*···*Cg*8^iv^	0.93	2.72	3.562 (4)	151
C19—H19*A*···*Cg*8^v^	0.93	2.78	3.643 (4)	154
C22—H22*A*···*Cg*7^vi^	0.93	2.72	3.567 (4)	151

Symmetry codes: (i) *x*, *y*, *z*+1; (ii) *x*, *y*, *z*−1; (iii) *x*+1, *y*, *z*; (iv) −*x*+1, −*y*+1, −*z*; (v) −*x*, −*y*+1, −*z*; (vi) −*x*+1, −*y*+1, −*z*+1.
